# Gestational and postnatal exposure to wildfire smoke and prolonged use of respiratory medications in early life

**DOI:** 10.1088/2752-5309/ad748c

**Published:** 2024-09-11

**Authors:** Hanna Jardel, Kristen M Rappazzo, Thomas J Luben, Corinna Keeler, Brooke S Staley, Cavin K Ward-Caviness, Cassandra R O’Lenick, Meghan E Rebuli, Yuzhi Xi, Michelle Hernandez, Ann Chelminski, Ilona Jaspers, Ana G Rappold, Radhika Dhingra

**Affiliations:** 1 Department of Epidemiology, Gillings School of Global Public Health, University of North Carolina Chapel Hill, Chapel Hill, NC, United States of America; 2 Oak Ridge Institute for Science and Education (ORISE) Predoctoral Fellow at United States Environmental Protection Agency (US EPA), Research Triangle Park, NC, United States of America; 3 U.S. Environmental Protection Agency, Office of Research and Development, Center for Public Health and Environmental Assessment, Research Triangle Park, NC, United States of America; 4 Center for Environmental Medicine, Asthma, and Lung Biology, University of North Carolina at Chapel Hill, Chapel Hill, NC, United States of America; 5 Department of Pediatrics, School of Medicine, University of North Carolina at Chapel Hill, Chapel Hill, NC, United States of America; 6 Department of Environmental Sciences and Engineering, Gillings School of Global Public Health, University of North Carolina at Chapel Hill, Chapel Hill, NC, United States of America; 7 Brody School of Medicine, East Carolina University, Greenville, NC, United States of America

**Keywords:** wildfire smoke, early childhood, respiratory medications, gestational exposure, developmental exposure

## Abstract

As wildfire frequency and severity increases, smoke exposures will cause increasingly more adverse respiratory effects. While acute respiratory effects of smoke exposure have been documented in children, longer term sequelae are largely unstudied. Our objective here was to examine the association between gestational and postnatal exposure to wildfire smoke and prolonged use of prescription medication for respiratory conditions in early childhood. Using Merative MarketScan claims data, we created cohorts of term children born in western states between 1 January 2010–31 December 2014 followed for at least three years. Using NOAA Hazard Mapping System data, we determined the average number of days a week that >25% of the population in a metropolitan statistical area (MSA) was covered by smoke within each exposure period. The exposure periods were defined by trimester and two 12 week postnatal periods. Medication use was based on respiratory indication (upper respiratory, lower respiratory, or any respiratory condition) and categorized into outcomes of prolonged use (⩾30 d use) (PU) and multiple prolonged uses (at least two prolonged uses) (MPU). We used logistic regression models with random intercepts for MSAs adjusted for child sex, birth season, and birth year. Associations differed by exposure period and respiratory outcome, with elevated risk of MPU of lower respiratory medications following exposure in the third trimester and the first 12 postnatal weeks (RR 1.15, 95% CI 0.98, 1.35; RR 1.21, 95% CI 1.05, 1.40, respectively). Exposure in the third trimester was associated with an increase in MPU of any respiratory among males infants only (male RR 1.22, 95% CI 1.00, 1.50; female RR 0.93, 95% CI 0.66, 1.31). Through novel use of prescription claims data, this work identifies critical developmental windows in the 3rd trimester and first 12 postnatal weeks during which environmental inhalational disaster events may impact longer-term respiratory health.

## 1. Background

Over the past decades, large and increasingly intense wildfires have produced smoke that negatively impacts air quality and public health in areas near and far from the fire [1]. Wildfire smoke is a complex mixture of gases and particulate matter, including fine particulate matter (PM_2.5_: particles with an aerodynamic diameter ⩽2.5 *µ*m) derived primarily from the combustion of biomass. However, as the area where houses are in or near wildland vegetation (wildland-urban interface), has increased, wildfires can also burn human-made structures and materials, potentially changing the composition of smoke [2–5]. Exposure to wildfire smoke has been linked to numerous acute health outcomes [6–10] including increased respiratory-related healthcare visits among children [11]. In an examination of longer-term childhood sequelae, our previous work showed shorter time to first use of respiratory medication in children with smoke exposures during developmental periods [12].

Studies of ambient PM_2.5_ have shown that both short- and long-term exposures can impact both lung function and development, respectively. Gestational and early infancy exposures to wildfire smoke may have particularly impactful effects, as lung development continues from gestation through adolescence [13, 14]. Postnatal exposure to wildfire smoke is associated with adolescent deficits in lung function and immune dysregulation in primates [15, 16]. Relatedly, exposure to PM_2.5_ in infancy has been associated with both immediate and lasting deficits in lung function [17–26]; however, it is unclear if exposure to wildfire smoke during developmental periods of life may impact children’s respiratory health severely enough to require prolonged medical intervention. Prolonged use of medications may indicate impaired respiratory health and potential susceptibility to developing chronic disease later in life [27].

Research on children’s respiratory health following wildfire smoke exposure using claims data has typically examined emergency department visits, asthma hospitalizations and outpatient visits for respiratory/asthma concerns immediately following exposure [28]. Longer term sequelae, such as prolonged infection or chronic wheeze, are not captured in these acute studies. Medication usage, particularly prolonged usage, can be used to examine rates of chronic or prolonged respiratory conditions that may result from exposure to wildfire smoke. For example, the number of refills of short-acting *β*-agonists was shown to be a reasonable indicator of asthma morbidity among adults [29]. Additionally, in a study of children 6–7 and 17–18 yrs old, increased self-reported medication use for respiratory symptoms was associated with duration of smell of fire smoke indoors and 5 d average PM_10_ concentrations during the height of a single fire season [30].

To further examine the relationship between respiratory outcomes and prenatal and early life exposure to wildfire smoke, we examined the prolonged use of medications indicated for respiratory conditions in young children. We differentiated between medications prescribed for upper respiratory and lower respiratory conditions and examined the potential modification of associations by sex.

## 2. Methods

### 2.1. Study design and population

We used data from the Truven MarketScan® Commercial Claims and Encounters Research Database (MarketScan) nationwide health insurance data. We included children born in six western states in the US, where wildFfires regularly occur, (California, Oregon, Washington, Idaho, Montana, Nevada) between 1st January 2010 and 31st December 2014. These states were chosen based on the burden estimations provided in Burke *et al* [31], which highlights these states as experiencing the highest burden.

Birthdates for live births were estimated using an algorithm to assess International Classification of Diseases (ICD) codes as described in Dhingra *et al* [12]. Briefly, we first determined if the infant or gestational parent (GP) ICD-9 codes included those for premature birth. Preterm births were excluded from the analysis. Because longer-term respiratory disease is more frequently observed in preterm infants due to their truncated respiratory development *in utero*, we assessed the feasibility of completing analyses on a cohort composed only of those born preterm. This sample had low numbers and failed to provide interpretable results and are thus not presented. If the infant was not born prematurely, we then determined if the GP had ICD-9 codes for long gestation—if so, we assigned the infant gestational age at birth as 42 weeks (*N* = 16). In the absence of the above codes, we assigned singleton birthed infants a gestational age at birth of 39 weeks and excluded all multiple births. Prescription use data consisted of name of the medication, claim date, and days of supply.

### 2.2. Outcome assessment: respiratory prescription use

Outcomes were defined as: (1) prolonged use (PU)—a child had one period of at least 30 d continuous use of a category of medication, and (2) multiple prolonged uses (MPU)—a child had at least two PUs. The characterization of a 30 d usage period as prolonged was determined in consultation with physicians—indicating a problem requiring sustained treatment.

We determined use periods for different categories of respiratory medication, accounting for washout (30 d) and grace periods (7 d) in defining each usage period. Categories of respiratory medication (including formulation) were validated by two physicians and include all respiratory medications, those prescribed for lower respiratory conditions, and those prescribed for upper respiratory conditions [12] (see etable e1). For example, albuterol is a common prescription medication used to treat asthma. It is included in the list of medications for lower respiratory conditions and in the ‘any medications’ list. Dexamethasone and prednisone were excluded as respiratory medications because the broad indications for systemic steroid prescription extend beyond respiratory conditions. Though antihistamines are indicated for both urticaria as well as rhinitis, these medications were retained in the upper respiratory category as they are more commonly prescribed for allergic rhinitis.

Note that the number of cases in the ‘any medications’ category is not simply a sum of the lower and upper respiratory outcome cases. For example, a participant could have used upper respiratory medications for <30 d followed by the period of use of lower respiratory medications for <30 d, and thus be counted as one case in the ‘any respiratory medications’ category only.

### 2.3. Exposure assessment: smoke-days

As described in Dhingra *et al* [12], we evaluated exposure as a continuous variable—average number of smoke-days per week in each trimester (T1: 280–197; T2: 196–99; T3: 98–1 d before estimated birth date), without restriction for wildfire seasons. T3 exposure estimates account for variable third trimester duration for each birth (e.g. those born at 42 weeks gestation are not assigned values of average exposure that assume 39 weeks gestation). We also examined exposure within two postnatal periods (PN1, PN2) defined as 1–84 d and 85–168 d post birth for PN1 and PN2, respectively, because crucial lung development occurs during this time [13].

Smoke exposure was classified based on Hazard Mapping System Fire and Smoke (HMS) data. The HMS smoke data derivation and validity have been described in detail elsewhere [32, 33]. Briefly, we used daily HMS data of smoke plume spatial extent across the coterminous US to classify each ZIP Code Tabulation Area (ZCTA) in the study area as experiencing a smoke-day, or not, based on the presence of smoke plume in a given ZCTA on a given day; a smoke plume of any density was counted as a smoke exposure is this binary assessment. Daily smoke-day classification for CBSAs were derived by using the ZCTA-metropolitan statistical area (MSA) crosswalk files developed by the US Office of Housing and Urban Development [34, 35] to generate population-weighted estimates of smoke exposure for each day for each MSA. An exposed day (i.e. smoke-day) was defined as each day for which an MSA met the exposure threshold of at least 25% of the population in an MSA experiencing wildfire smoke. Alternate smoke-day thresholds, 0% population affected by smoke (i.e. any smoke occurred in MSA regardless of estimated population exposure) and 50% population affected by smoke, are examined as sensitivity analyses. There is inherent uncertainty in using plume data to assess wildfire smoke exposure, as areas adjacent to the visible plume are likely exposed to wildfire pollutants. Because the visible wildfire plumes likely underrepresent the true exposure in a given MSA to an uncertain degree, we chose, a priori, to use multiple lower thresholds (0%, 25% and 50%) expecting that 25% and 50% are likely to be more representative of the population’s true wildfire smoke exposure. In our data, there is a difference in exposure (any smoke exposure during pregnancy) for a relatively small subset of children (∼2000) between the 25th and 50th percentile thresholds and marginal changes in results. Thus, for the ease of presentation, we chose to present the 25% threshold in the main text. For each infant, the average smoke-days per week in a given exposure period was determined by summing days exposed during that exposure period (e.g. T3) and dividing by the duration of the exposure period in weeks (13 weeks for T1 and T2, 12 weeks for PN1 and PN2, and T3 weeks specific to that infant calculated based on completed T3 weeks at delivery). This was done to allow for comparison across different lengths of gestation, and for consistency with the previous analysis [12]. Risk Ratios (RR) for medication use periods are given per increase of one average smoke-day per week in that exposure period. An increase of one average smoke-day per week during full trimesters (13 weeks) translates to 13 smoke-days exposure, regardless of how those days are distributed (e.g. one 13 d fire or 2 week-long fires).

### 2.4. Analytical cohorts

We created a total of three analytical cohorts: 3 yr cohorts for each exposure period (gestational T1–T3 exposures, PN1 exposures, and PN2 exposures) to examine potential differences in respiratory health in infancy compared to a longer duration in early life with the understanding that respiratory development continues through this period. Each cohort was restricted to non-preterm births and at least 3 yr enrolled after the end of the exposure period (gestation, PN1, or PN2), then to birth MSAs with at least 200 eligible births. The postnatal exposure period cohorts included enrollment time that started 7 d after the exposure period ended (e.g. PN1 enrollment time starts at 91 d post birth) (efigures e1 and e2).

### 2.5. Statistical analysis

We used log binomial regression models with random intercepts for birth MSA to estimate RR and 95% confidence intervals (95% CI) for PU and MPU per one additional weekly smoke-day for each exposure window. Each exposure period was examined separately.

Covariates in models included dichotomous indicator of child sex (male/female), meteorological birth season (1st December–28th/29th February, 1st March –31st May, 1st June–31st August, 1st September–30th November for Winter, Spring, Summer, Fall, respectively), and year of birth. Sex was used as a proxy for hormones present in early life with the understanding that physician assigned sex at birth generally corresponds to hormone distributions. No other demographic information was available in MarketScan data.

The primary analysis examined dichotomous PU and MPU of respiratory medication in relation to 1 additional average smoke-day per week among the 3 yr cohorts. The analyses also examined the potential for effect measure modification (EMM) by sex, as previous analyses [12] and our knowledge of lung development [13, 14] indicates potential for different responses by sex. For this, we included an interaction between sex and the respective exposure in adjusted models. Secondary analyses included repeating all procedures with 1 yr cohorts and with adjustments including MSA level percent of population with annual incomes below the federal poverty line as defined by the American Community Survey estimates for 2009–2013 [36].

All data processing and analysis was completed with SAS v9.4 (Cary, North Carolina) and R (Vienna, Austria) [37] packages *lme4* [38], and *lmtest* [39].

### 2.6. Missing data

In this analysis there were no records in the analytical cohorts that were missing covariate data.

## 3. Results

### 3.1. Descriptions

Within the 3 yr gestational cohort (*N* = 35, 065), there were 3588 (10.2%) individuals with PU of any respiratory medication(s) (table 1). Of these, 379 (1.1%) used only upper respiratory medications, and 2560 (71.3%) used only lower respiratory medications. Within the same cohort, there were 774 (2.2%) individuals who had MPU of any respiratory medication; of these, 76 (0.2%) used upper respiratory medications only and 635 (1.8%) used lower respiratory medications only. Detailed breakdowns of characteristics across the remaining five cohorts are shown in etables e2–e6. The greatest number of births were in MSAs within Los Angeles, Portland, and Seattle (etable e7). The overall exposure distribution shows about equal minima, medians, and maxima with a broader distribution interquartile range in T1 (etable e8, efigure e3).

**Table 1.** Characteristics for 3 yr gestational exposure cohort^a^ by prolonged use^b^ and multiple prolonged uses^c^ of respiratory medication use.

	Upper respiratory	Lower respiratory	Any respiratory^d^	Overall
*n*	378	2556	3582	35 022
Sex (%)				
Female	216 (57.1)	1528 (59.8)	2122 (59.2)	18 101 (51.7)
Male	162 (42.9)	1028 (40.2)	1460 (40.8)	16 921 (48.3)
Birth season (%)^e^				
Spring	115 (30.4)	715 (28.0)	998 (27.9)	9712 (27.7)
Summer	101 (26.7)	725 (28.4)	1007 (28.1)	9784 (27.9)
Fall	93 (24.6)	645 (25.2)	907 (25.3)	8696 (24.8)
Winter	69 (18.3)	471 (18.4)	670 (18.7)	6830 (19.5)
Year of birth (%)				
2010	110 (29.1)	740 (29.0)	1002 (28.0)	8564 (24.5)
2011	89 (23.5)	539 (21.1)	777 (21.7)	7371 (21.0)
2012	74 (19.6)	453 (17.7)	654 (18.3)	6871 (19.6)
2013	55 (14.6)	475 (18.6)	657 (18.3)	6611 (18.9)
2014	50 (13.2)	349 (13.7)	492 (13.7)	5605 (16.0)
*n*	76	635	774	35 022
Sex (%)				
Female	49 (64.5)	420 (66.1)	496 (64.1)	18 101 (51.7)
Male	27 (35.5)	215 (33.9)	278 (35.9)	16 921 (48.3)
Birth season (%)				
Spring	19 (25.0)	153 (24.1)	183 (23.6)	9712 (27.7)
Summer	25 (32.9)	175 (27.6)	217 (28.0)	9784 (27.9)
Fall	18 (23.7)	172 (27.1)	221 (28.6)	8696 (24.8)
Winter	14 (18.4)	135 (21.3)	153 (19.8)	6830 (19.5)
Year of birth (%)				
2010	30 (39.5)	192 (30.2)	247 (31.9)	8564 (24.5)
2011	17 (22.4)	128 (20.2)	164 (21.2)	7371 (21.0)
2012	9 (11.8)	113 (17.8)	134 (17.3)	6871 (19.6)
2013	10 (13.2)	118 (18.6)	138 (17.8)	6611 (18.9)
2014	10 (13.2)	84 (13.2)	91 (11.8)	5605 (16.0)

Overall exposure distribution of average smoke-days in exposure period				

Gestational exposures	Minimum	Median	Interquartile range	Maximum

Trimester 1	0.00	0.08	(0.00, 0.50)	4.75
Trimester 2	0.00	0.07	(0.00, 0.29)	4.64
Trimester 3	0.00	0.08	(0.00, 0.31)	4.77

^a^ Cohort composed of those followed for first 3 yr of life.

^b^ At least one period of 30 d continuous use of prescription respiratory medication for respiratory conditions (not including systemic steroid use).

^c^ At least two periods of 30 d continuous use of prescription respiratory medication for respiratory conditions (not including systemic steroid use).

^d^ Any respiratory outcome is not a sum of lower respiratory and lower respiratory medication outcomes (see Methods section for more detail).

^e^ Meteorological birth season (1st December–28th/29thFebruary, 1st March–31st May, 1st June–31st August, 1st September–30th November for Winter, Spring, Summer, Fall, respectively).

In the 3 yr gestational cohort, those with PU of all categories of respiratory medication were more likely to be female in comparison to the overall cohort and the overall proportion of females was greater than males (table 1). This pattern was exaggerated with MPU of all categories of respiratory medication. Birth season and year of birth were generally well balanced between those in all categories of outcome (PU and MPU of all medication categories) and the overall cohort.

### 3.2. Primary analysis

#### 3.2.1. 3 yr cohorts (prolonged use and MPU outcomes)

**Figure 1.**
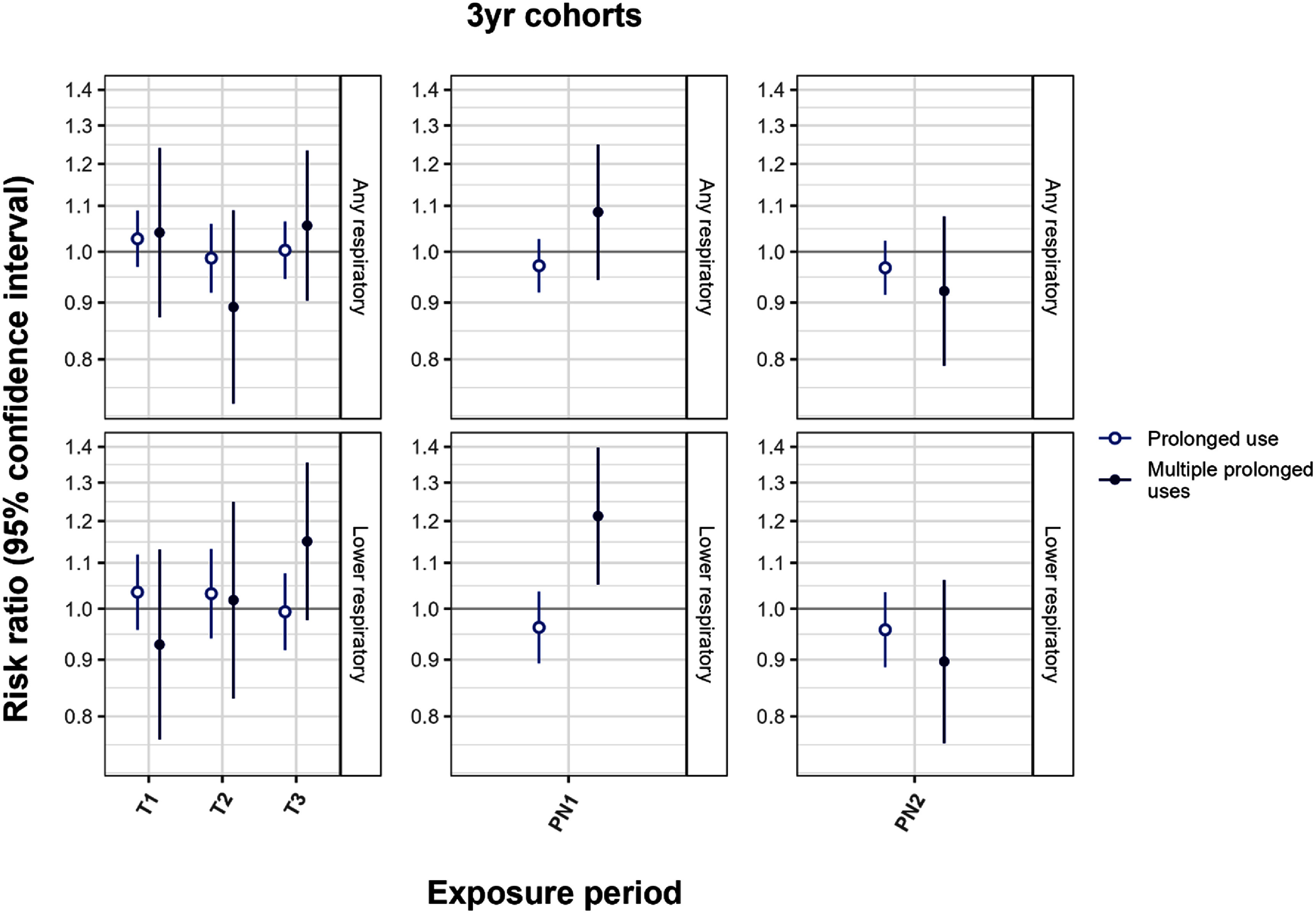
Risk ratios for prolonged use and multiple prolonged uses of respiratory medication with an increase of 1 smoke-day per week in 3 yr cohorts by outcome and medication category. Adjusted for child sex, meteorological birth season, and year of birth. The top row shows results for all medications, the bottom row shows results for lower respiratory medications; columns correspond to exposure periods. Underlying numbers in etable e9.

For the gestational exposure periods and PU outcome, all point estimates were approximately null (figure 1, etable e9 1 yr cohort results efigure e4). When considering the MPU outcome there was more variability in the results. For any respiratory medications, the point estimates were essentially null. For lower respiratory medication, we observed null effects with T1, T2 and PN2 exposures, while RRs with T3 and PN1 exposures were increased (RR = 1.15, 95% CI: 0.98, 1.35; RR = 1.21, 95% CI: 1.05, 1.40, respectively). Due to small numbers in the 3 yr cohorts, models for upper respiratory medications did not converge and are not discussed.

### 3.3. Effect measure modification and Sex-specific analyses

#### 3.3.1. 3 yr cohorts (prolonged use and MPU outcomes)

**Figure 2.**
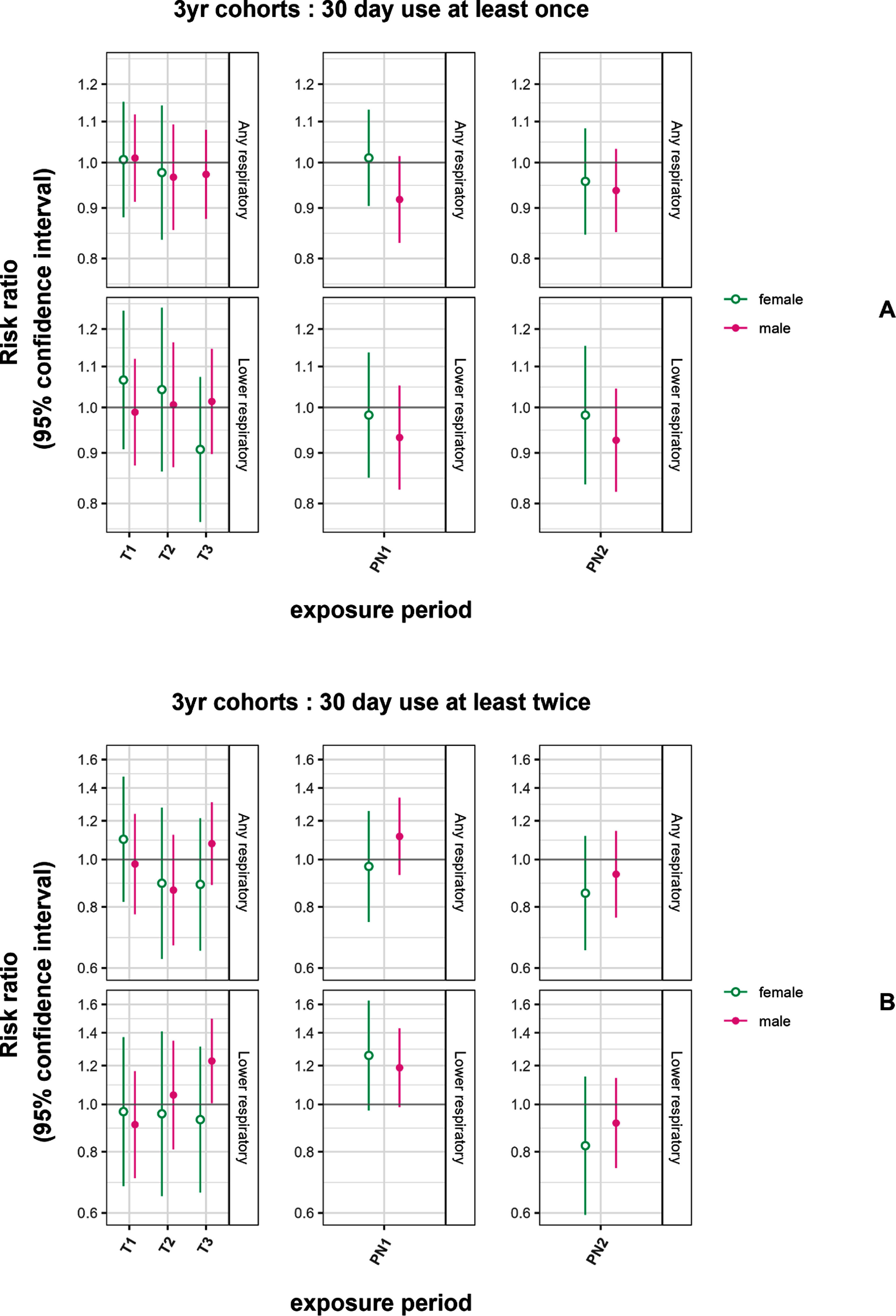
Sex-specific risk ratios for prolonged use and multiple prolonged uses of respiratory medication with an increase of 1 smoke-day per week in 3 yr cohorts by outcome and medication category. Adjusted for child sex, meteorological birth season, and year of birth. The top panel (A) shows results for all medications, the bottom panel (B) shows results for lower respiratory medications; columns correspond to exposure periods.

EMM analysis provided evidence to support effect measure modification by sex only within the PN1 exposure period with the PU outcome for the ‘any respiratory’ medications category, with a ‘protective’ effect for males (stratified analysis: female RR = 1.01, 95% CI: 0.90, 1.13; male RR = 0.92, 95% CI: 0.83, 1.02) (figure 2(a), etables e10 and e11 1 yr-cohort results shown in efigure e5).

When examining the MPU outcome, male and female RR were largely similar and null with few exceptions (figure 2(b)). An elevated RR MPU estimate for lower respiratory medications and T3 exposure for males only (male RR = 1.22 95% CI: 1.00, 1.50; females RR = 0.93, 95% CI: 0.66, 1.31). Both male and female RR for lower respiratory outcome and PN1 exposure were similarly elevated, as in the overall models (male RR = 1.19 95% CI: 0.99, 1.42; females RR = 1.26, 95% CI: 0.97, 1.63).

Overall results across cohorts were robust to changes in exposure threshold (efigures e6(a) and e7(b), etable e12), and to adjustment for percent of population living in poverty line (etable e13).

## 4. Discussion

In this study of prolonged use of respiratory medications and smoke-day exposures per week during and immediately following gestation, we observed an increase in the repeated, prolonged use of lower respiratory medications in the first 3 yr after exposure among children who were exposed to wildfire smoke during the 3rd trimester and the first 12 weeks after birth (PN1). An increase in the repeated, prolonged use of lower respiratory medications was associated with increased smoke-day exposures among male children in the 3rd trimester and first 12 postnatal weeks, and among female children in the first 12 postnatal weeks only. The potential sex specific differences in risk were found to be consistent with a previous assessment of associations between postnatal smoke-days exposure and time to first upper respiratory prescription use by Dhingra *et al* [12]. This study did not show consistent associations between gestational exposures with both prolonged use and MPU of respiratory medication of any category. While we observed a potential protective effect of smoke exposure among male children exposed in the first 12 weeks after birth for at least one prolonged use of any respiratory medication (PU), this effect was not retained when considering at least two episodes of prolonged use of any respiratory medication.

This is the first study examining prolonged respiratory prescription use during early life in response to wildland fire smoke exposures during development. Because we cannot compare the results from this study directly to prior work, we refer to the knowledge of biological process and epidemiologic studies concerning similar exposure (e.g. ambient air pollution and biomass burning) and related outcomes. Lung development occurs in stages and continues through adolescence; thus, exposure to wildfire smoke at different times may cause different responses. Whereas exposure to wildfire smoke in early development may lead to changes in cellular differentiation and structure [40], exposure during later development may cause functional changes [41, 42]. Additionally, lung development is sex-specific in that phases occur at different times for the sexes and contribute to morphological differences (e.g. females develop surfactant sooner, which is suspected to lead to development of smaller airways) [14], thus exposures at sex-specific critical windows may elicit sex-specific responses [12], which may be partially due to differences in susceptibility to infections [43].

A literature review of early life exposure to biomass cooking fuel emissions found that acute respiratory infections before 5 yr of age were associated with biomass fuel emission exposure [44]. Respiratory infections in young children are common and often require use of respiratory medications to either directly address the infection or manage the physiological effects of infection; though we lack access to this data, respiratory infections may well comprise the basis for most respiratory prescriptions in our dataset. Dutta *et al* [2021, 45] found that higher postnatal exposures to PM were associated with higher airway resistance. Additionally, Shao *et al* [2020, 46] found an association between infant exposure to coal mine fire-emitted fine PM and greater incidence of antibiotic dispensations. While the present study did not include antibiotics among its prescriptions of interest, the exposures are similar and the studies indicate a change in lung health in response to postnatal exposure. In the context of previous literature, the present study indicates there may be greater sensitivity to exposure to wildfire smoke in the postnatal period, as opposed to less direct prenatal routes: particles passed through the gestational parent’s biological systems [47–49], exposure to the gestational parent’s compensation mechanisms (i.e. oxidative stress and inflammation)[50, 51] or placental dysfunction as a result of gestational parent exposure [52].

Previous literature on ambient particulate matter suggests that gestational wildfire smoke exposure may affect conditions such as transient tachypnea of the newborn, asphyxia, wheezing, recurrent broncho-pulmonary infections, and prolonged cough (⩾1 month) within 1st year of life, deficits in lung function in early infancy as well as early childhood (⩽5), and early (0–6 yrs) childhood asthma [17–22, 25, 26]. These outcomes, while indicative of declines in respiratory health, may not warrant prolonged pharmaceutical intervention.

This study has several limitations. Outcome misclassification may have occurred because we were not able to account for compliance with medication; however, the data source provides certainty that medications were both prescribed and filled. We were additionally unable to categorize use of systemic steroids, thus our results may reflect attenuated associations if many children were prescribed only these medications for respiratory conditions. As with all studies concerning outcomes among neonates and infants, this study likely suffers from live birth bias [53]. Wildfire has repeatedly been shown to increase the likelihood of preterm birth [54, 55], and preterm children are more likely, varying considerably by the degree of prematurity, to have childhood respiratory conditions due to their truncated time *in utero* [56]. Consequently, preterm children represent an important subgroup who are likely etiologically different from their term counterparts and more vulnerable to pre- and peri-natal wildfire smoke exposures. Due to small numbers, however, we were unable to study this vulnerable subgroup. Exposure measurement error may have occurred through a few mechanisms. MSAs are relatively large and not every part of an MSA would experience the exact same exposure. This exposure measure does not account for differences in smoke plume density beyond its presence or absence. Additionally, we assumed that MSA at birth was the same MSA throughout gestation and post-birth exposure periods. We believe this is a reasonable assumption, as residential mobility studied during gestation is relatively low [57–61], but movement out of the MSA of birth at any point of the exposure period may lead to exposure misclassification. Lack of behavioral data prevented us from accounting for variations in indoor/outdoor time or the presence of indoor air filtration systems. Due to the lack of demographic data, we were not able to account for individual socioeconomic factors or parental stress which may act as modifiers of this association. This study also does not account for potential mediation or modification through birth defects or parental health wherein compromised health in early development may lead to greater susceptibility to wildfire smoke impacts in children. Future studies with more levels of demographic data could explore these factors as modifiers to improve elucidation of potential exposure-response pathways. MarketScan data is representative of medication use by people living in the study area who are enrolled in private health insurance plans. Caution should be exercised in extending these results to populations without private health insurance or rural areas. In addition, while here we were interested in investigating early life exposures, there is potential for lifetime cumulative exposures to produce different associations.

Despite these limitations, this study contributes to the literature with by aiming to examine prolonged respiratory conditions in relation to wildfire smoke exposure, and do so considering varying windows of exposure and outcome periods. Additionally, it includes a broad geographic range and accounts for differences in baseline risk between MSAs with random intercept models.

## 5. Conclusions

Overall, this works suggest that are potential long-term benefits to reducing infant exposure to wildfire smoke. Future research could examine lung health in cohorts of infants exposed at different critical windows followed into adulthood to investigate the potential for long-lasting impacts of gestational and early life exposure to wildfire smoke while accounting for sociodemographic characteristics.

## Data Availability

Merative MarketScan® Research Database, a proprietary claims dataset on which this work is based, is available through Truven (www.merative.com/real-world-evidence). The environmental data from the NOAA Hazards Mapping System Fire and Smoke (HMS) that support the conclusions of this article are available in the U.S. EPA Environmental Dataset Gateway (www.ospo.noaa.gov/Products/land/hms.html#maps).
